# Patient engagement in care: A scoping review of recently validated tools assessing patients' and healthcare professionals' preferences and experience

**DOI:** 10.1111/hex.13344

**Published:** 2021-08-16

**Authors:** Nathalie Clavel, Jesseca Paquette, Vincent Dumez, Claudio Del Grande, Djahanchah Philip (Sacha) Ghadiri, Marie‐Pascale Pomey, Louise Normandin

**Affiliations:** ^1^ Ingram School of Nursing McGill University Montreal Quebec Canada; ^2^ Health Innovation and Evaluation Hub University of Montreal Hospital Center Montreal Quebec Canada; ^3^ Center of Excellence on Partnership with Patients and the Public University of Montreal Montreal Quebec Canada; ^4^ Department of Health Management, Evaluation and Policy, School of Public Health University of Montreal Montreal Quebec Canada; ^5^ Department of Management HEC MONTRÉAL Montreal Quebec Canada

**Keywords:** clinical care, healthcare professionals, patient engagement, patients, scoping review, validated tools

## Abstract

**Background:**

Patient engagement in care is a priority and a key component of clinical practice. Different approaches to care have been introduced to foster patient engagement. There is a lack of a recent review on tools for assessing the main concepts and dimensions related to patient engagement in care.

**Objective:**

Our scoping review sought to map and summarize recently validated tools for assessing various concepts and dimensions of patient engagement in care.

**Search Strategy:**

A scoping review of recent peer‐reviewed articles describing tools that assess preferences in and experience with patient engagement in care was conducted in four databases (Ovid Medline, Ovid EMBASE, Cochrane Database of Systematic Reviews, CINAHL‐EBSCO). We adopted a broad definition based on the main concepts of patient engagement in care: patient‐centredness, empowerment, shared decision‐making and partnership in care.

**Main Results:**

Of 2161 articles found, 16, each describing a different tool, were included and analysed. Shared decision‐making and patient‐centredness are the two main concepts evaluated, often simultaneously in most of the tools. Only four scales measure patient‐centredness, empowerment and shared decision‐making at the same time, but no tool measures the core dimensions of partnership in care. Most of the tools did not include patients in their development or validation or just consulted them during the validation phase.

**Discussion and Conclusion:**

There is no tool coconstructed with patients from development to validation, which can be used to assess the main concepts and dimensions of patient engagement in care at the same time.

**Patient and Public Contribution:**

This manuscript was prepared with a patient expert who is one of the authors. Vincent Dumez, who is a patient expert and codirector of the Center of Excellence on Partnership with Patients and the Public, has contributed to the preparation of the manuscript.

## 1 INTRODUCTION

Patient engagement in health policies, healthcare planning and improvement and direct care is recognized as a cornerstone of quality and safety.^1^ Engaging patients in care has become a priority and a key component of clinical practice in many countries around the world.^2^ Evidence suggests that engaging patients can help (re)shape their care and treatment in ways that fit their needs and preferences, ultimately resulting in improved outcomes.^3^, ^4^, ^5^, ^6^ Over the last few decades, various approaches to care have been introduced in clinical practice to foster the integration of patient engagement into the delivery of healthcare.

In the 1990s, the patient‐centred care approach gradually replaced the medical paternalism that has dominated healthcare for decades.^7^ The patient‐centred care model involves integrating patients' needs and preferences into the delivery of care, ‘moving away from a logic of “care to patients” towards one of “care for patients”’.^7^ Patient‐centred care is based on a patient‐oriented perspective of care that includes what patients consider important for their life project.^7^

In the last two decades, collaborative approaches have emerged, moving towards a logic of ‘care with patients’. For example, shared decision‐making encourages patients to take part in decisions on their care,^8^, ^9^ while self‐management or patient education approaches seek to strengthen patients' knowledge and skills to better empower them in their care process.^10^, ^11^ More recently, the ‘partnership in care’ approach has considered patients as full‐fledged members of healthcare teams.^12^ This new model builds on aspects of each care approach, including the integration of patient needs and preferences into care delivery, the participation of patients in care decisions, the coconstruction of a care plan with them and the development of patients' capacities to manage their own care.^13^ A previous empirical study on patient partnership from the patients‐as‐partners perspective was able to define partnership in care as the proactive efforts by patients to fill the gap between their preferences or expectations in the care relationship and what they experience during a consultation with a healthcare professional (HCP).^12^

All these approaches to care are part of a continuum of engagement, from consultation to partnership in care, that reflects the increasingly important role played by patients in their own care.^1^

As patient engagement in care has become a clinical standard in healthcare settings, a growing number of scales have been developed in recent decades for effective quality improvement. Previous reviews have identified, synthesized and appraised the tools used to assess some dimensions of care/approaches to care. A recent systematic review by Philipps et al.^14^ focuses on studies published between 2005 and 2014 that described tools for assessing patient participation in healthcare. Their review only includes tools that measure approaches related to self‐management and decision‐making, without including tools that assess patient‐centred care. Conversely, a review by Ree et al.^15^ synthetizes information on patient‐centred care tools, with a special focus on how patients are involved in the care. Lastly, a review by Jerofke‐Owen et al.^16^ identifies and appraises tools that measure self‐reported patients' preferences in engagement, without considering tools that assess patient experience in engagement or HCPs' preferences in and experience with engaging patients. However, as patient engagement is part of the care relationship between patients and HCPs, it is important to identify the various tools used to assess both patients' and HCPs' preferences and experience in this area. In addition, the literature lacks a broad review of the recently validated scales used to assess the central dimensions on which the different approaches to care (from consultation to partnership in care) are based and that coexist in clinical practices.

The objective of this scoping review was therefore to map and summarize recently validated tools that assess patient engagement in care. To this end, several specific objectives were pursued: (1) to identify tools used to assess both patients' and HCPs' preferences in and/or experiences with patient engagement in care; (2) to summarize the evidence on recently validated tools that assess various concepts and dimensions of patient engagement in care; and (3) to report on various characteristics of scale development and validation (patient involvement, reliability of the scales).

## 2 METHODS

### 2.1 Conceptual framework

In this review, we adopted a broad definition of patient engagement in care that reflects the continuum of such engagement and the coexistence of different approaches to enhancing it. The definitions proposed here are based on Castro et al.'s^17^ conceptual analysis of the main concepts related to patient engagement in care: patient‐centred care, empowerment and shared decision‐making. These concepts refer to different approaches to patient engagement in care, and together, they form the higher concept of partnership in care. According to Pomey et al.,^18^ the concept of partnership in care is an approach that considers the patient as ‘a caregiver of herself and, as such, a genuine member of the treatment team, endowed with competencies and limitations just like any other member of the team’. We believe that the concept of partnership can be summarized by all the dimensions on which each approach to care is based,^7^ including individualized care, empathy, interpersonal trust, communication, experiential knowledge and self‐care. A summary of these concepts' definitions and dimensions is presented in Table 1.

**Table 1:** Definitions of the main concepts related to patient engagement in care, adapted from Castro et al.^17^

Concept	Partnership in care main dimensions: Individualized care, empathy, interpersonal trust, communication, experiential knowledge and self‐care^7^		
	Patient centredness	Shared decision‐making	Empowerment
Definition	‘Biopsychosocial approach and attitude that aims to deliver care that is respectful and individualized. It implies the individual participation of the patient and is built on a relationship of mutual trust, sensitivity, empathy and shared knowledge’^17^	‘Patient's rights and opportunities to influence and engage in the decision‐making about his care through a dialogue attuned to his preferences, potential and a combination of his experiential and the professional's expert knowledge’^17^	‘Process that enables patients to exert more influence over their individual health by increasing their capacities to gain more control over issues they themselves define as important’^17^
Main dimensions	‐ Person‐centred care/climate ‐ Individualized care (considering needs, values and preferences) ‐ Empathy ‐ Therapeutic alliance ‐ Interpersonal trust	‐ Shared decision‐making ‐ Patient–provider communication ‐ Experiential knowledge	‐ Self‐care/self‐management ‐ Patient education ‐ Patient enablement ‐ Patient activation ‐ Experiential knowledge

We used the continuum of patient involvement in research^18^ to identify the level of patient involvement in tool development and validation. We considered three levels of involvement: (1) consultation of patients, which refers to asking for patients' input during the validation of the tool; (2) collaboration with patients, which corresponds to involving them in the selection and wording of items; and (3) partnership, which refers to coconstructing the tool with patients, from its development to its validation.

### 2.2 Review approach

We conducted a scoping review to map recent evidence on validated scales for assessing patient engagement in care.^19^ We chose to conduct a narrative synthesis of the literature to describe the major characteristics of the tools, including the measurement objective, the concepts and dimensions assessed, the clinical context of utilisation and the development and validation characteristics of the tools. We followed the PRISMA extension for scoping reviews to apply a systematic approach when conducting the review and reporting the results.^20^

### 2.3 Searches and screening

We searched for articles published between 2014 and 2021 in four major health and social science databases: Ovid MEDLINE, Ovid EMBASE, the Cochrane database of systematic reviews and EBSCO‐CINAHL. We decided to search for articles published starting from 2014 since a previous systematic review by Philipps et al.^14^ searched for articles published from 2005 to 2013 that described tools for measuring patient participation in care. As Philips et al.^14^ adopted a rather broad definition of patient engagement in care (shared decision‐making, self‐care and patients having self‐knowledge), we wanted to ensure that our review would not duplicate any previous reviews on this topic. The initial search was conducted on 19 January 2021. The search terms used are presented in Table 2. These correspond to the six major concepts searched for, related to patient, engagement, assessment, scale, clinical care and validation. Our search strategy was limited to published and peer‐reviewed literature and we did not conduct any searches in the grey literature. The search strategies developed for Ovid MEDLINE are available in File S1. The search strategies developed for the three other databases are available upon request. Two reviewers (N. C. and J. P.) independently screened articles based on their titles and abstracts. A pilot round was conducted with 50 references to verify the authors' (N. C. and J. P.) agreement on the inclusion and exclusion criteria before performing a full screening of the rest of the articles.

**Table 2:** Search terms

Patient	Engagement	Assessment	Scale	Clinical	Validation
Patient	Engagement	Assessment	Scale	Clinical care	Validation
User	Involvement	Measurement	Tool	Care	Psychometry
Client	Participation	Evaluation	Survey	Clinical level	Reliability
	Centred care		Questionnaire		Validity
	Shared decision‐making				Factor analysis
	Empowerment				
	Self‐management				
	Patient education				
	Partnership				
	Activation				
	Enablement				

### 2.4 Inclusion and exclusion criteria

We included both original and review articles describing the development and validation of scales assessing patient engagement in adult inpatient or outpatient care. The inclusion and exclusion criteria are detailed in Table 3.

**Table 3:** Inclusion and exclusion criteria

Inclusion criteria	Exclusion criteria
Original or review articles describing the development and validation of scales assessing patient engagement in care	Published in a language other than English or French
Studies describing tools used in adult inpatient or outpatient care (all clinical settings)	Studies describing tools developed to be used with specific chronic or acute disease patients
Studies published between 2014 and 2021	Studies describing tools used in paediatric care with children or adolescents
	The reviewers could not access the full text (the authors were contacted, but did not respond)

### 2.5 Extraction methods

Data from the included articles were charted on an extraction grid (File S2) according to the following categories: (1) the general presentation of the scales (e.g., first author, year of publication, title of the article and journal of publication), (2) the specific characteristics of the scales (e.g., name of the scale; measurement objective; patients', family members' or health professionals' perspective; preferences in or experience with patient engagement; context of utilisation; number of items; and scale measurement) and (3) the methodological aspects of tool development and validation (e.g., level of patient involvement in tool development or validation and reliability of the scale).

Furthermore, the extraction grid included Castro et al.'s^17^ three main concepts related to patient engagement in care: (1) patient‐centred care, (2) empowerment and (3) shared decision‐making. One author (J. P.) classified each tool's main factors/dimensions or items according to the corresponding concepts, and a second author (N. C.) reviewed each component attribution.

## 3 RESULTS

### 3.1 Search results

**Figure 1:**
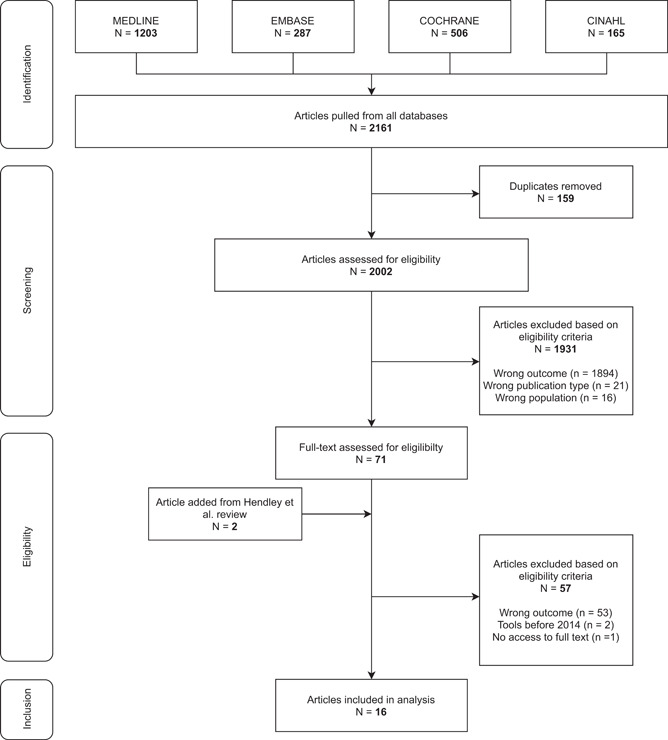
Flowchart of the study selection process

Our initial search yielded a total of 2161 articles. After removing duplicates, a total of 2002 articles were screened. This process resulted in a total of 71 full‐text articles to be assessed for eligibility. One of them, a review of surveys for measuring patient‐centred care in the hospital setting,^21^ included two tools published after 2014: the Family Inventory of Needs^22^ and the Person‐Centred Climate Questionnaire‐Family Version (PCQ‐F).^23^ Consequently, we have added the two articles corresponding to the development and validation of these tools to assess them against our full eligibility criteria. Following reviews of 73 full‐text articles, 16 articles fulfilled the inclusion criteria. The PRISMA flow diagram is shown in Figure 1.

### 3.2 Study characteristics

We identified a total of 16 articles, representing 16 different tools, based on the eligibility criteria for assessing patient engagement in care. All the findings are presented in Tables 4 and 5. The data extraction table containing all the information that we extracted from the tools can also be accessed from File S2. The included studies were published between 2015 and 2020, and all use a quantitative design based on psychometric data analysis to describe the development and validation of tools assessing patient engagement in care. The studies were from eight different countries around the world: the United States (*n* = 5), China (*n* = 2), Denmark (*n* = 2), Italy (*n* = 2), Sweden (*n* = 2), Belgium (*n* = 1), the Netherlands (*n* = 1) and Taiwan (*n* = 1). The included studies were published in 12 different journals, some of which were represented more than once: the *Journal of Advanced Nursing* (*n* = 2), the *Journal of Nursing Care Quality* (*n* = 2), the *Journal of Nursing Management* (*n* = 2) and *Patient Education & Counseling* (*n* = 2). The scales contain between 9 and 55 items, most of which are rated on a five‐point Likert scale (56%, *n* = 9).

**Table 4:** Tools included in the scoping review assessing the concepts and dimensions related to patient engagement in care (2014–2021)

Author and year of publication Name of the scale	Perspective and preferences in or experience with care measured	Patient involvement in tool development	Patient involvement in tool validation	Concepts assessed
Berg et al. (2020)^24^	Patients' perspective, experience of engagement	No	Yes	Patient‐centredness, shared decision‐making
Patient Participation Questionnaire				
Casu et al. (2019)^25^	Patients' perspective, experience of engagement	Not reported	Not reported	Patient‐centredness, shared decision‐making
Patient–Professional Interaction Questionnaire				
Cramm and Nieboer (2018)^26^	Patients' perspective, experience of engagement	Yes	No	Patient‐centredness, shared decision‐making
36‐Item patient‐centred primary care instrument				
Fridberg et al. (2020)^27^	Patients' perspective, Experience of engagement	No	Yes	Patient‐centredness, shared decision‐making, empowerment
Generic Person‐Centred Care Questionnaire				
Greene et al. (2017)^28^	Health professionals' perspective, experience of engagement	Not reported	Not reported	Patient‐centredness, empowerment
No name				
Gremigni et al. (2016)^29^	Health professionals' perspective, experience of engagement	No	No	Patient‐centredness, shared decision‐making
Provider–Patient Relationship Questionnaire				
Huang et al. (2018)^30^	Health professionals' perspective, experience of engagement	No	No	Patient‐centredness, shared decision‐making, empowerment
Five‐Dimension Patient‐Centred Innovation Questionnaire				
Jerofke and Weiss (2016)^31^	Patients' perspective, experience of engagement	No	Yes	Patient‐centredness, shared decision‐making
Patient Perceptions of Patient‐Empowering Nurse Behaviours Scale				
Lindhal et al. (2015)^23^	Family members' perspective, experience of engagement	Not reported	Not reported	Patient‐centredness
Person‐Centred Climate Questionnaire‐Family Version				
Malfait et al. (2016)^32^	Health professionals' perspective, both experience and preferences for engagement	No	Yes	Shared decision‐making
Patient Participation Culture Tool for Healthcare Workers				
Riegel et al. (2018)^33^	Patients' perspective, experience of engagement	No	No	Empowerment
Self‐care of chronic illness inventory (SC‐CII or Sky)				
Stichler and Pelletier (2020)^34^	Patients' perspective, experience of engagement	Yes	No	Shared decision‐making, empowerment
Patient Empowerment, Engagement, and Activation Survey				
Wang et al. (2019)^35^	Patients' perspective, experience of engagement	No	No	Shared decision‐making, empowerment
Inpatients' Involvement in Medication Safety Scale				
Wu et al. (2020)^36^	Patients' perspective, experience of engagement	No	Yes	Patient‐centredness, shared decision‐making, empowerment
Patient Engagement in Health Care Questionnaire				
Yen et al. (2020)^37^	Patients and family members' perspective, preferences for engagement	Yes	No	Patient‐centredness, shared decision‐making, empowerment
WeCares				
Zachariae et al. (2015)^38^	Health professionals' perspective, experience of engagement	No	No	Patient‐centredness, shared decision‐making
Student and physician self‐efficacy in patient‐centredness				

**Table 5:** Characteristics of the tools included in the scoping review, including context of utilisation, number of items, scale measurement and index of reliability

Authors, year and name of the scale	Journal	Context of utilisation	Number of items	Scale measurement	Index of reliability
Berg et al. (2020)^24^	*European Journal of Cardiovascular Nursing*	Any ward within the departments of cardiology, thoracic surgery and heart and lung transplantation	16 Items	4‐Point numeric rating scales	Cronbach's *α* = .89
Patient Participation Questionnaire					
Casu et al. (2019)^25^	*Patient Education & Counseling*	Hospital settings	16 Items	5‐Point scale	Cronbach's *α* = .95
Patient–Professional Interaction Questionnaire					
Cramm et al. (2018)^26^	*BMC Family Practice*	Primary care setting (patients with multimorbidity)	36 Items	5‐Point scale	Cronbach's *α* = .89
36‐Item patient‐centred primary care instrument					
Fridberg et al. (2020)^27^	*BMC Health Services Research*	In‐ and outpatient care units	18 Items	4‐Point rating scale	Person separation index = 0.85
Generic Person‐Centred Care Questionnaire					
Greene et al. (2017)^28^	*Healthcare*	Primary care—Fairview Health Services	9 Items	1–5 Scale	Cronbach's *α* = .73
No name					
Gremigni et al. (2016)^29^	*Patient Education & Counseling*	Hospital settings	16 Items	5‐Point scale	Cronbach's *α* = .90
Provider–Patient Relationship Questionnaire					
Huang et al. (2018)^30^	*Journal of Nursing Management*	Hospital settings—clinical services	55 Items	5‐Point Likert scale	Cronbach's *α* = .94
Five‐Dimension Patient‐Centred Innovation Questionnaire					
Jerofke et al. (2016)^31^	*Journal of Advanced Nursing*	Clinical settings	42 Items (22 Items in short form)	11‐Point Likert scale	Cronbach's *α* = .98 (long from), .97 (short form)
Patient Perceptions of Patient‐Empowering Nurse Behaviours Scale					
Lindhal et al. (2015)^23^	*Scandinavian Journal of Caring Sciences*	Emergency department	17 Items	6‐Point Likert‐type scale	Cronbach's *α* = .93
Person‐Centred Climate Questionnaire‐Family Version					
Malfait et al. (2016)^32^	*International Journal of Nursing Studies*	General and university hospital wards	52 Items	4‐Point Likert scale	Cronbach's *α* = .92
Patient Participation Culture Tool for Healthcare Workers					
Riegel et al. (2018)^33^	*Journal of Advanced Nursing*	Inpatient and outpatient settings (patients with chronic illness)	20 Items	5‐Point ordinal response scale	Reliability index for multidimensional scales = 0.71
Self‐care of chronic illness inventory					
Stichler et al. (2020)^34^	*Journal of Nursing Care Quality*	Acute care and specialty hospitals (behavioural health and women and newborns)	21 Items	5‐Point Likert‐like response set	Cronbach's *α* = .88
Patient Empowerment, Engagement, and Activation Survey					
Wang et al. (2019)^35^	*Journal of Nursing Management*	Inpatients from a tertiary hospital—clinical nursing	23 Items	Not reported (only for content validity: Likert 5‐grade score)	Cronbach's *α* = .916
Inpatients' Involvement in Medication Safety Scale					
Wu et al. (2020)^36^	*Journal of Nursing Care Quality*	General hospitals	33 Items	5‐Point Likert scale	Cronbach's *α* = .928
Patient Engagement in Health Care Questionnaire					
Yen et al. (2020)^37^	*Joint Commission Journal on Quality & Patient Safety*	Intensive care units	13 Items	4‐Point Likert scale and 5‐point Likert scale	Cronbach *α* for each subscale = .7116–.866
WeCares					
Zachariae et al. (2015)^38^	*BMC Medical Education*	Medical school and hospitals	27 Items	5‐Point Likert scale	Cronbach's *α* = .92 and .94 for medical students (different sample); .95 for physicians
Student and physician self‐efficacy in patient‐centredness					

### 3.3 Perspectives on patient engagement as measured by the tools

Nearly all tools measure experience in patient engagement (88%, *n* = 14), most of which comes from the patients' perspective (56%, *n* = 9).^24^, ^25^, ^26^, ^27^, ^31^, ^33^, ^34^, ^35^, ^36^ Tools that inquire about health professionals' perspectives are also mostly based on their own experience in engaging patients (25%, *n* = 4),^28^, ^29^, ^32^, ^38^ but one tool, PaCT‐HCW,^32^ evaluates both preferences in and experience with patient engagement. Finally, two tools assess how family members perceived engagement in care: the PCQ‐F tool ^23^ measures their experience with engagement and the WeCares tool^37^ evaluates their preferences.

### 3.4 Concepts and dimensions assessed with the tools

We categorized the dimensions assessed in each tool into the main concepts of engagement, which include patient centredness, empowerment and shared decision‐making. The results reveal that the two main concepts evaluated in the included scales are shared decision‐making (81%, *n* = 13)^24^, ^25^, ^26^, ^27^, ^29^, ^30^, ^32^, ^34^, ^35^, ^36^, ^37^, ^38^ and patient centredness (75%, *n* = 12),^23^, ^24^, ^25^, ^26^, ^27^, ^28^, ^29^, ^30^, ^31^, ^36^, ^37^, ^38^ while empowerment is present in 50% (*n* = 8) of the included tools.^27^, ^28^, ^30^, ^33^, ^34^, ^35^, ^36^, ^37^

Nine tools evaluate two concepts simultaneously.^24^, ^25^, ^26^, ^28^, ^29^, ^31^, ^34^, ^35^, ^38^ Of these, six evaluate (37%) both patient‐centredness and shared decision‐making. These tools, including the Patient–Professional Interaction Questionnaire,^25^ the Provider–Patient Relationship Questionnaire,^29^ the Patient Participation Questionnaire,^24^ the Patient‐Centred Primary Care instrument,^26^ the Patient Perceptions of Patient‐Empowering Nurse Behaviours Scale^31^ and the Student and physician self‐efficacy in patient‐centeredness,^38^ assess common dimensions of patient‐centredness (empathy/human approach, individualized care/patients' preferences, trust and information exchange) and shared decision‐making (patient involvement in care decisions, patients encouraged to ask questions and effective communication). Three of these tools measure the patient experience of engagement from the patient's perspective^24^, ^25^, ^26^ and two from the HCP's point of view.^29^, ^38^ Four tools have been developed to be used in inpatient care,^24^, ^25^, ^29^, ^38^ while one has been validated in primary care settings.^26^ The three main concepts of patient engagement in care are evaluated in four of the scales (25%): the Generic Person‐Centred Care Questionnaire,^27^ the Five‐Dimension Patient‐Centred Innovation Questionnaire,^30^ the Patient Engagement in Health Care Questionnaire^36^ and the WeCares survey.^37^ Three of them measure the patient experience of engagement,^27^, ^30^, ^36^ three assess the patient and/or the family perspective^27^, ^36^, ^37^ and one evaluates the point of view of the HCPs.^30^ Three tools have been validated in inpatient care settings^30^, ^36^, ^37^ and one is a generic tool that can be used in both inpatient and outpatient care.^27^ Finally, three scales (18%) only evaluate a single concept.^23^, ^32^, ^33^

**Figure 2:**
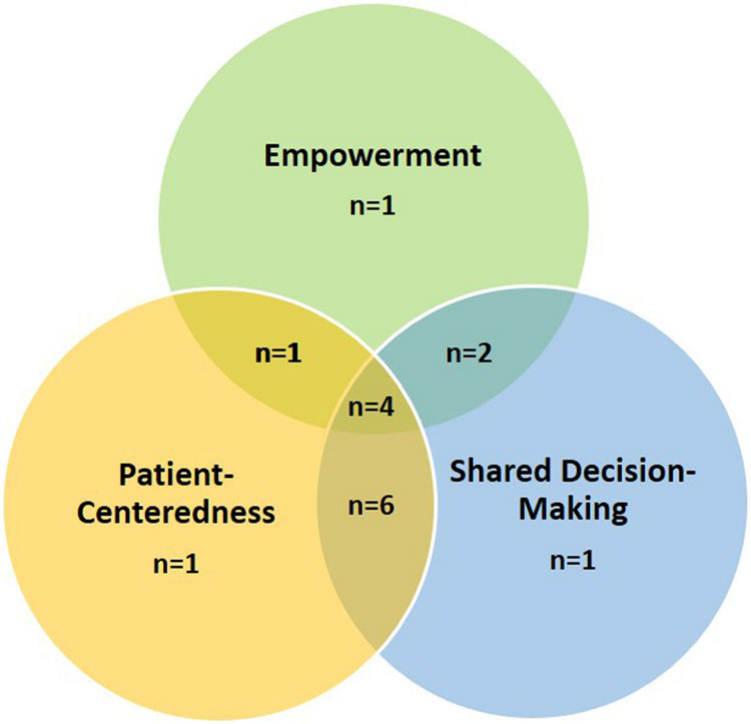
Venn diagram presenting the number of tools assessing one or several concepts of patient engagement in care

Figure 2 shows the number of tools assessing one or several concepts of patient engagement in care.

### 3.5 Involvement of patients in tool development or validation

A total of eight tools (50%) did not include patients in their development or validation or did not report having involved patients, and eight were partly developed with patients. For five of them (31%), the involvement of patients consisted mostly of consulting them to validate the tool, including with respect to face or content validity.^24^, ^27^, ^31^, ^32^, ^36^ Three tools (19%) were developed in collaboration with patients for item development through focus groups or in‐depth interviews or by including patients in expert panels.^26^, ^34^, ^37^ Nevertheless, none of the tools were coconstructed in partnership with patients from item development to the validation phase.

Table 4 presents details on perspectives on patient engagement as measured by the tools, the concepts and dimensions assessed and patient involvement in tool development and validation.

### 3.6 Context of utilisation of the tools

Most of the tools (75%, *n* = 12) have been validated in inpatient care,^23^, ^24^, ^25^, ^29^, ^30^, ^31^, ^32^, ^34^, ^35^, ^36^, ^37^, ^38^ while a few (13%, *n* = 2) were developed for use in outpatient care.^26^, ^28^ Only two tools have been validated in both inpatient and outpatient clinical settings.^27^, ^33^ Figure 2 shows information on the context of utilisation of the tools included in our scoping review.

### 3.7 Psychometric properties of the validated tools

Lastly, in terms of psychometric properties, the reported reliability indices of the tools (Cronbach's *α*, the reliability index for multidimensional scales and the person separation index) were between 0.71 and 0.98. Based on these coefficients, we consider the reliability of the tools to be moderate to excellent.^14^

Table 5 presents information on various characteristics of the tools, including the context of their use, the number of items, the measurement scale and psychometric properties (index of reliability).

## 4 DISCUSSION

To the best of our knowledge, this is the first systematic scoping review to have identified and summarized recently validated tools assessing various concepts and dimensions of patient engagement in care from both the patients' and HCPs' perspectives. Unlike the systematic review by Jerofke and Weiss,^31^ which identified instruments measuring patient preferences regarding engagement in healthcare, our broad review included scales that assessed preferences in or experience with patient engagement from the patients' and HCPs' perspectives.

This scoping review identified and summarized 16 studies that reported on the development and validation of 16 tools assessing patient engagement in care. Nearly all of these tools measure experience with patient engagement and a few measure preferences in patient engagement or both preferences in and experience with engagement simultaneously. The results also show that most of the tools were validated in inpatient care settings and did not involve patients in their development or only consulted them during the validation phase. We identified four important findings concerning the development and validation of the tools included in our scoping review. First, a few tools simultaneously measure some dimensions related to all of the concepts of patient engagement. Second, tools that assess preferences regarding patient engagement in care are relatively scarce. Third, our scoping review has also shown that no tool has been developed in coconstruction with patients, from development to validation. Lastly, very few of the tools were generic, meaning that they could be used in various contexts of care.

### 4.1 Dimensions measured in tools assessing patient engagement in care

Most of the tools assess dimensions related to one or two concepts of patient engagement (patient centredness or empowerment or shared decision‐making), and only a few tools simultaneously measure some dimensions related to all of the concepts of patient engagement.^27^, ^30^, ^36^, ^37^ Furthermore, we found that no tool simultaneously assesses the core dimensions of the partnership in care presented in our conceptual framework (individualized care, empathy, interpersonal trust, communication, experiential knowledge and self‐care).^7^

Tools assessing preferences regarding patient engagement in care are relatively scarce. Two out of 16 scales only or partly measured preferences in engagement: One from the HCPs' point of view and the other from both the patients' and family members' perspective. Only one tool, PaCT‐HCW,^32^ evaluates both preferences in and experience with patient engagement, but only from the HCPs' point of view. The assessment of patient engagement in care should consider both preferences in and experience with such engagement. From our previous empirical work, which consisted of understanding how patients perceived the partnership in care, we have shown that partnership can be understood as the fit between patients' preferences or expectations for engagement in their care and their experience with such engagement with their HCP.^12^ Consequently, measurement of partnership in care could be achieved using a tool capable of assessing the congruence between patients' needs or preferences in their relationship with their HCPs and their perceptions of those interactions.

### 4.2 Context of tool development, validation and utilization

Our scoping review has also shown that no tool has been developed in coconstruction with patients, from development to validation. For only three tools, patients collaborated in the tool's development, in terms of item selection, through expert panels or focus groups or through in‐depth interviews. There is a consensus that patients should be more actively involved in research to ensure that studies focus on issues that are relevant to them.^39^, ^40^ It seems particularly important to co‐construct tools with patients when the tools are designed for them. Furthermore, developing a tool to assess patient engagement in care requires a bottom‐up or coconstruction approach with patients and HCPs^41^ to identify important dimensions of patient engagement based on the patients' conceptions of being engaged in care and the HCPs' perspectives of engaging patients.

Lastly, very few of the tools were generic. Most of the tools were validated in specific hospital settings, and only two tools were validated in both inpatient and outpatient settings. As most of the tools were not generic, they cannot be used in various contexts of care, in inpatient or outpatient clinical settings, or with patients who have various chronic or acute diseases. As patient engagement in care has become a clinical standard in healthcare settings, it would be important to develop a generic tool that can be used to measure patient engagement in various clinical contexts. This could help monitor the situation and improve patient–HCP relationships, which is one of the core dimensions of quality.^42^

### 4.3 Strengths and limitations

The main strengths of this scoping review are its broad search strategy, which was based on a broad conceptual framework. This allowed us to include various tools assessing the various concepts related to patient engagement in care, and their identification of the levels of patient involvement in tool development and validation. We also used systematic methods to conduct our scoping review, as two reviewers independently screened articles and extracted data,^43^ and we complied with the PRISMA extension for scoping reviews.^20^ Lastly, we mapped our findings with the broad conceptual framework used to develop our search strategies.

There are two main limitations to our scoping review that may have limited our ability to identify all the relevant tools. First, we only searched for articles published in English or French, which could have led to missing publications in other languages. However, this bias appears to be limited since there is no evidence of systematic bias from using only English‐language articles in systematic reviews.^44^ Second, we decided to limit our search to articles published starting from 2014 because a previous systematic review by Phillips et al.^14^ covered articles published from 2003 to 2014 on tools for measuring patient participation in care. Philipps et al.'s^14^ conceptual framework on patient engagement was not strictly identical to the one that we adopted for our scoping review. However, in their systematic review, Phillips et al.^14^ included two of the three main concepts of patient engagement (shared decision‐making and self‐care) that we included in our own conceptual framework. For this reason, we may have missed some relevant tools measuring patient‐centred care by applying this limit.

### 4.4 Conclusion and implications for clinical practice and research

A growing number of tools have been developed and validated in recent years for assessing patient engagement in care. This scoping review offers a broad identification and description of various tools assessing central concepts in the various approaches to care (patient centredness, empowerment and shared decision‐making) that coexist in clinical practices and settings. No tool offers an exhaustive assessment of the various concepts and dimensions related to both preferences in and experience with engagement in care. However, four tools stand out because they measure the three major concepts of patient engagement in care (patient centredness, empowerment and shared decision‐making).^27^, ^30^, ^36^, ^37^ Three of them, and the most recently developed, have involved patients in their tool development or validation.^27^, ^36^, ^37^ Involving patients in the development and validation of tools that assess the quality of care can contribute to uncovering complementary aspects of quality that are not necessarily considered as important by researchers or HCPs. Engaging patients in healthcare research is largely encouraged as it adds value, quality and appropriateness to the research process and outputs.^45^ This practice is even more essential when developing a tool that specifically seeks to measure the key dimensions of patient engagement in care. Lastly, the tools that were found in this scoping review only assess preferences in or experience with engagement in care. Nevertheless, measurement of engagement should focus on both patients' preferences in and experience with engagement as a means to appropriately identify the gap between their expectations and experience with their HCPs. A tool measuring this gap in patient engagement could help HCPs to improve the way they interact with patients during consultations, thus improving the quality of care.

The partnership in care approach, which builds on and integrates various approaches to care and dimensions of patient engagement, has started to be applied in clinical practices and different clinical settings. We therefore argue that there is a need for an exhaustive tool that (1) assesses the core dimensions of the partnership in care, (2) measures both preferences in and experiences with engagement, (3) evaluates patients', family members' and HCPs' perspectives to improve partnership practices between patients and their HCPs and (4) has been developed in partnership with patients.

## CONFLICT OF INTERESTS

The authors declare that there are no conflict of interests.

## AUTHOR CONTRIBUTIONS

Nathalie Clavel is the first author of this manuscript. All authors contributed to at least some component of the scoping review and/or manuscript. Nathalie Clavel shaped all aspects of the study design with feedback from Jesseca Paquette and Marie‐Pascale Pomey. Nathalie Clavel and Jesseca Paquette screened independently the titles and abstracts of articles found in the databases and extracted data from full‐text articles. Nathalie Clavel and Jesseca Paquette wrote the manuscript and Vincent Dumez, Claudio Del Grande, Djahanchah Philip (Sacha) Ghadiri, Marie‐Pascale Pomey and Louise Normandin gave substantial suggestions and feedback. All authors have read and approved the final manuscript.

## Data Availability

The data that support the findings of this study are available in the Supporting Information Material of this article.

## References

[1] Carman KL , Dardess P , Maurer M , et al. Patient and family engagement: a framework for understanding the elements and developing interventions and policies. Health Aff. 2013;32(2):223‐231. 10.1377/hlthaff.2012.1133 23381514

[2] Armstrong MJ , Mullins CD , Gronseth GS , Gagliardi AR . Impact of patient involvement on clinical practice guideline development: a parallel group study. Implement Sci. 2018;13(1):55. 29661195 10.1186/s13012-018-0745-6 PMC5902835

[3] Bodenheimer T , Lorig K , Holman H , Grumbach K . Patient self‐management of chronic disease in primary care. JAMA. 2002;288(19):2469‐2475. 12435261 10.1001/jama.288.19.2469

[4] Crawford MJ , Rutter D , Manley C , et al. Systematic review of involving patients in the planning and development of health care. BMJ. 2002;325(7375):1263. 12458240 10.1136/bmj.325.7375.1263 PMC136920

[5] Trotti A , Colevas AD , Setser A , Basch E . Patient‐reported outcomes and the evolution of adverse event reporting in oncology. J Clin Oncol. 2007;25(32):5121‐5127. 17991931 10.1200/JCO.2007.12.4784

[6] Hibbard JH , Greene J , Tusler M . Improving the outcomes of disease management by tailoring care to the patient's level of activation. Am J Manag Care. 2009;15(6):353‐360. 19514801

[7] Dumez V , Pomey MP . From medical paternalism to care partnerships: a logical evolution over several decades. In: Macmillan P , ed. Patient Engagement: How Patient‐Provider Partnerships Transform Healthcare Organizations. Palgrave Macmillan; 2019:9‐16.

[8] Gravel K , Légaré F , Graham ID . Barriers and facilitators to implementing shared decision‐making in clinical practice: a systematic review of health professionals' perceptions. Implement Sci. 2006;1:16. 16899124 10.1186/1748-5908-1-16 PMC1586024

[9] Elwyn G , Frosch D , Thomson R , et al. Shared decision making: a model for clinical practice. J Gen Intern Med. 2012;27(10):1361‐1367. 22618581 10.1007/s11606-012-2077-6 PMC3445676

[10] Foster G , Taylor SJ , Eldridge SE , Ramsay J , Griffiths CJ . Self‐management education programmes by lay leaders for people with chronic conditions. Cochrane Database Syst Rev. 2007;(4):Cd005108. 17943839 10.1002/14651858.CD005108.pub2

[11] Grady PA , Gough LL . Self‐management: a comprehensive approach to management of chronic conditions. Am J Public Health. 2014;104(8):e25‐e31. 24922170 10.2105/AJPH.2014.302041 PMC4103232

[12] Pomey M , Ghadiri DP , Karazivan P , Fernandez N , Clavel N . Patients as partners: a qualitative study of patients' engagement in their health care. PLoS One. 2015;10(4):e0122499. 25856569 10.1371/journal.pone.0122499 PMC4391791

[13] Karazivan P , Dumez V , Flora L , et al. The patient‐as‐partner approach in health care: a conceptual framework for a necessary transition. Acad Med. 2015;90(4):437‐441. 25607943 10.1097/ACM.0000000000000603

[14] Phillips NM , Street M , Haesler E . A systematic review of reliable and valid tools for the measurement of patient participation in healthcare. BMJ Qual Saf. 2016;25(2):110‐117. 10.1136/bmjqs-2015-004357 26415751

[15] Ree E , Wiig S , Manser T , Storm M . How is patient involvement measured in patient centeredness scales for health professionals? A systematic review of their measurement properties and content. BMC Health Serv Res. 2019;19(1):12. 30621682 10.1186/s12913-018-3798-y PMC6323701

[16] Jerofke‐Owen T , Garnier‐Villarreal M , Fial A , Tobiano G . Systematic review of psychometric properties of instruments measuring patient preferences for engagement in health care. J Adv Nurs. 2020;76(8):1988‐2004. 10.1111/jan.14402 32350898

[17] Castro EM , Van Regenmortel T , Vanhaecht K , Sermeus W , Van Hecke A . Patient empowerment, patient participation and patient‐centeredness in hospital care: a concept analysis based on a literature review. Patient Educ Couns. 2016;99(12):1923‐1939. 27450481 10.1016/j.pec.2016.07.026

[18] Pomey M , Flora L , Karazivan P , et al. Le “Montreal Model”: enjeux du partenariat relationnel entre patients et professionnels de la santé. Sante Publique. 2015;HS(S1):41‐50. 26168616

[19] Munn Z , Peters MDJ , Stern C , Tufanaru C , McArthur A , Aromataris E . Systematic review or scoping review? Guidance for authors when choosing between a systematic or scoping review approach. BMC Med Res Methodol. 2018;18(1):143. 30453902 10.1186/s12874-018-0611-x PMC6245623

[20] Tricco AC , Lillie E , Zarin W , et al. PRISMA extension for scoping reviews (PRISMA‐ScR): checklist and explanation. Ann Intern Med. 2018;169(7):467‐473. 30178033 10.7326/M18-0850

[21] Handley SC , Bell S , Nembhard IM . A systematic review of surveys for measuring patient‐centered care in the hospital setting. Med Care. 2021;59(3):228‐237. 33229897 10.1097/MLR.0000000000001474 PMC7878319

[22] Catlin A , Ford M , Maloney C . Determining family needs on an oncology hospital unit using interview, art, and survey. Clin Nurs Res. 2016;25(2):209‐231. 25862492 10.1177/1054773815578806

[23] Lindahl J , Elmqvist C , Thulesius H , Edvardsson D . Psychometric evaluation of the Swedish language Person‐centred Climate Questionnaire‐family version. Scand J Caring Sci. 2015;29(4):859‐864. 25648407 10.1111/scs.12198

[24] Berg SK , Færch J , Cromhout PF , et al. Questionnaire measuring patient participation in health care: scale development and psychometric evaluation. Eur J Cardiovasc Nurs. 2020;19(7):600‐608. 32324044 10.1177/1474515120913809

[25] Casu G , Gremigni P , Sommaruga M . The Patient‐Professional Interaction Questionnaire (PPIQ) to assess patient centered care from the patient's perspective. Patient Educ Couns. 2019;102(1):126‐133. 30098906 10.1016/j.pec.2018.08.006

[26] Cramm JM , Nieboer AP . Validation of an instrument for the assessment of patient‐centred care among patients with multimorbidity in the primary care setting: the 36‐item patient‐centred primary care instrument. BMC Fam Pract. 2018;19(1):143. 30153809 10.1186/s12875-018-0832-4 PMC6114899

[27] Fridberg H , Wallin L , Wallengren C , Kottorp A , Forsman H , Tistad M . Development and evaluation of the measurement properties of a generic questionnaire measuring patient perceptions of person‐centred care. BMC Health Serv Res. 2020;20(1):960. 33081770 10.1186/s12913-020-05770-w PMC7574493

[28] Greene J , Sacks RM , Hibbard JH , Overton V . How much do clinicians support patient self‐management? The development of a measure to assess clinician self‐management support. Healthc (Amst). 2017;5(1‐2):34‐39. 27594306 10.1016/j.hjdsi.2016.05.007

[29] Gremigni P , Casu G , Sommaruga M . Dealing with patients in healthcare: a self‐assessment tool. Patient Educ Couns. 2016;99(6):1046‐1053. 26851160 10.1016/j.pec.2016.01.015

[30] Huang C‐Y , Weng R‐H , Wu T‐C , et al. Developing and testing the patient‐centred innovation questionnaire for hospital nurses. J Nurs Manag. 2018;26(2):227‐237. 28960600 10.1111/jonm.12539

[31] Jerofke T , Weiss M . Development and psychometric analysis of the Patient Perceptions of Patient‐Empowering Nurse Behaviours Scale (PPPNBS). J Adv Nurs. 2016;72(11):2923‐2936. 27377456 10.1111/jan.13060

[32] Malfait S , Eeckloo K , Van Daele J , Van Hecke A . The Patient Participation Culture Tool for healthcare workers (PaCT‐HCW) on general hospital wards: a development and psychometric validation study. Int J Nurs Stud. 2016;61:187‐197. 27372433 10.1016/j.ijnurstu.2016.05.015

[33] Riegel B , Barbaranelli C , Sethares KA , et al. Development and initial testing of the self‐care of chronic illness inventory. J Adv Nurs. 2018;74(10):2465‐2476. 29943401 10.1111/jan.13775

[34] Stichler JF , Pelletier LR . Psychometric testing of a patient empowerment, engagement, and activation survey. J Nurs Care Qual. 2020;35(4):E49‐E57. 31821184 10.1097/NCQ.0000000000000452

[35] Wang B‐H , Zhang J‐H , Zhang J , Zhu Q , Yan Q‐Y . The development and psychometric testing of Inpatients' Involvement in Medication Safety Scale (IIMSS). J Nurs Manag. 2019;27(8):1648‐1654. 31444838 10.1111/jonm.12852

[36] Wu Q , Ye X , Wu Y , Zhao L . Development and psychometric evaluation of the patient engagement in Health Care Questionnaire. J Nurs Care Qual. 2020;35(3):E35‐E40. 32433156 10.1097/NCQ.0000000000000439

[37] Yen PY , Lehmann LS , Snyder J , et al. Development and validation of WeCares, a survey instrument to assess hospitalized patients' and family members' “Willingness to Engage in Your Care and Safety”. Jt Comm J Qual Patient Saf. 2020;46(10):565‐572. 32883579 10.1016/j.jcjq.2020.07.002 PMC9472245

[38] Zachariae R , O'Connor M , Lassesen B , et al. The self‐efficacy in patient‐centeredness questionnaire—a new measure of medical student and physician confidence in exhibiting patient‐centered behaviors. BMC Med Educ. 2015;15:150. 26374729 10.1186/s12909-015-0427-x PMC4572680

[39] Domecq JP , Prutsky G , Elraiyah T . Patient engagement in research: a systematic review. BMC Health Serv Res. 2014;14:89. 24568690 10.1186/1472-6963-14-89 PMC3938901

[40] Boivin A , L'espérance A , Gauvin FP , et al. Patient and public engagement in research and health system decision making: a systematic review of evaluation tools. Health Expect. 2018;21:1075‐1084. 30062858 10.1111/hex.12804 PMC6250878

[41] Graffigna G , Barello S , Bonanomi A , Lozza E . Measuring patient engagement: development and psychometric properties of the Patient Health Engagement (PHE) Scale. Front Psychol. 2015;6:274. 25870566 10.3389/fpsyg.2015.00274 PMC4376060

[42] Elwyn G , Lloyd A , Joseph‐Williams N , et al. Option grids: shared decision making made easier. Patient Educ Couns. 2013;90(2):207‐212. 22854227 10.1016/j.pec.2012.06.036

[43] Peters MDJ , Godfrey CM , Khalil H , McInerney P , Parker D , Soares CB . Guidance for conducting systematic scoping reviews. JBI Evidence Implementation. 2015;13(3):141‐146. 10.1097/XEB.0000000000000050 26134548

[44] Morrison A , Polisena J , Husereau D , et al. The effect of English‐language restriction on systematic review‐based meta‐analyses: a systematic review of empirical studies. Int J Technol Assess Health Care. 2012;28(2):138‐144. 22559755 10.1017/S0266462312000086

[45] Martineau JT , Minyaoui A , Boivin A . Partnering with patients in healthcare research: a scoping review of ethical issues, challenges, and recommendations for practice. BMC Med Ethics. 2020;21(1):34. 32393230 10.1186/s12910-020-0460-0 PMC7216517

