# Supplementary material for: Patient engagement in care: A scoping review of recently validated tools assessing patients' and healthcare professionals' preferences and experience
Source: Health Expect. 2021 Aug 16;24(6):1924–35. doi: 10.1111/hex.13344 (PMC8628592; doi:10.1111/hex.13344)
Supplement: Supplementary file 1 [file HEX-24-1924-s001.pdf]

Supplementary file 1. Search strategy developed for Ovid-MEDLINE

#	**Search terms and strategy**
**1**	(((engag* or incolv* or participat* or shar*) adj2 decision*) or centered-care or centered care or centred-care or partner* or enabl* or activat* or empower* or self-management* or self management or patient education).ab,ti.
**2**	(patient* or user* or client* or consumer*).ab,ti.
**3**	(tool* or scale* or survey* or questionnaire*).ab,ti.
**4**	(assess* or evaluat* or measur*).ab,ti.
**5**	(validat* or psychometric* or factor analysis or validity or reliability).af
**6**	(clinical care or care or clinical level*).ab,ti.
**7**	Patient participation/
**8**	*Outcome Assessment, Health Care/ or *Process Assessment, Health Care/
**9**	*Patient-Centered Care/
**10**	*Self Care/
**11**	*Decision Making, Shared/
**12**	1 or 7 or 9 or 10 or 11
**13**	4 or 8
**14**	2 and 3 and 5 and 6 and 12 and 13
**15**	Limit 14 to (English language and yr="2014-Current")
**16**	Limit 15 to (adaptive clinical trial or address or autobiography or bibliography or biography or case reports or clinical conference or clinical study or clinical trial, all or clinical trial, phase I or clinical trial, phase ii or clinical trial, phase iii or clinical trial, phase iv or clinical trial, veterinary or clinical trials, veterinary as topic or clinical trial protocol or clinical trial protocols as topic or clinical trial or comment or congress or consensus development conference or consensus development conference, nih or controlled clinical trial or dataset or dictionary or editorial or equivalence trial or "expression of concern" or government publication or guideline or historical article or interactive tutorial or interview or lecture or legal case or legislation or letter or news or newspaper article or observational study, veterinary or personal narrative or pragmatic clinical trial or preprint or published erratum or randomized controlled trial or randomized controlled trial, veterinary or "research support, American recovery and reinvestment act" or research support, nih, extramural or research support, nih, intramural or research support, non us gov’t or research support, us gov’t, non phs or research support, us gov’t, phs or retracted publication or "retraction of publication" or "scientific integrity review" or technical report or video-audio media or webcast)
